# The effect of WeChat-based brisk walking on kinesiophobia in patients after percutaneous coronary intervention: a randomised controlled trial

**DOI:** 10.3389/fcvm.2025.1416356

**Published:** 2025-04-29

**Authors:** Bo-Ya Wan, Ce Zhou, Xing Sun, Feng-Min Yu, Mei-Xia Liu, Jia-Yuan Zhang, Qi Li, Li Zhang

**Affiliations:** ^1^ Hebei Key Laboratory of Clinical Pharmacy, Department of Cardiology, Hebei General Hospital, Shijiazhuang, Hebei, China; ^2^ Department of Cardiology, The Second People's Hospital of Hengshui, Hengshui, Hebei, China; ^3^ Department of Cardiology, Affiliated Hospital of Hebei University of Engineering, Handan, Hebei, China; ^4^ Nursing Department, BAO DING NO1 General Hospital Baoding, Hebei, China

**Keywords:** cardiac rehabilitation, WeChat, brisk walking, kinesiophobia, nursing

## Abstract

**Aim:**

This study aimed to evaluate the benefit of a home-based cardiac rehabilitation (CR) programme with telemonitoring guidance on kinesiophobia in patients with percutaneous coronary intervention (PCI) discharged from hospital. It also aimed to explore the effectiveness of this programme on self-efficacy and cardiorespiratory endurance using a randomised controlled trial.

**Design:**

This study was a single-blind, prospective randomised controlled trial.

**Methods:**

Patients who underwent PCI at our hospital were enrolled. The intervention group (IG) performed WeChat-based brisk walking and the control group (CG) received the usual care, including advice to remain physically active. All patients underwent cardiopulmonary exercise testing to assess their peak oxygen uptake (peak VO_2_) at baseline and after a 12-week intervention period. The main outcome indicator was kinesiophobia. Secondary outcomes included exercise self-efficacy, cardiorespiratory endurance (i.e., peak VO_2_), and major adverse cardiovascular events.

**Results:**

A total of 137 patients were enrolled in this study between 1 February 2023 and 31 October 2023. Of them, 130 patients successfully completed a 12-week WeChat-based brisk walking CR programme. After 12 weeks of intervention, the Tampa Scale for Kinesiophobia Heart scores in the IG decreased significantly more than in the CG and pre-IG. The IG's Self-Efficacy for Exercise scores were higher than those of the CG and pre-IG. In addition, the increase in peak VO_2_ was larger in the IG than in the CG.

**Conclusion:**

The WeChat-based brisk walking programme is beneficial for reducing kinesiophobia by increasing exercise self-efficacy in patients after PCI; it also helps to improve cardiopulmonary endurance. WeChat-based brisk walking is feasible and acceptable.

## 1 Introduction

Coronary heart disease (CHD) is the most prevalent cardiovascular disease. It is a common condition and is the leading cause of mortality and disability in adults (1). This burden disproportionately affects low- and middle-income countries, contributing to approximately 7 million deaths and 129 million disability-adjusted life years annually (2). The primary medical treatment for CHD is percutaneous coronary intervention (PCI) (3), with 10,142,266 patient cases reported in China in 2021 (4, 5). Cardiac rehabilitation (CR) (6) is an important therapeutic principle for secondary prevention in patients with CHD, which reduces all-cause mortality caused by various coronary events (7–9). Cardiac rehabilitation reduces hospitalisations and increases the quality of life in PCI (10). In addition, exercise-based CR can improve physical capacity and peak oxygen consumption in patients with CHD (11). However, studies show that <20% of patients participate in CR, and the compliance is low. Kinesiophobia is an important obstacle to taking part in CR and one of the main reasons for those who fail to carry on exercising.

Regular physical activity plays a significant role in reducing mortality rates among patients with CHD. For patients who undergo postoperative PCI, non-compliance with exercise therapy after discharge increases the likelihood of a poor prognosis and readmission. Several national and international clinical guidelines recommend that post-PCI patients participate in exercise rehabilitation to improve functional status, exercise capacity, and quality of life.

Kinesiophobia was first identified by Kori, and it was mostly applied to research on agoraphobia in people with chronic pain. Reports on the incidence of kinesiophobia in patients with CHD are in the range of 20%–70% at home and abroad. A Chinese cross-sectional study (12) showed that 41.76% of 376 patients with CHD experienced kinesiophobia. According to a Polish study (13), >70% of people with CHD experience kinesiophobia. Kinesiophobia affects 45% of individuals with CR and dramatically lowers the rate of CR visits (14). In CR, the identification and treatment of kinesiophobia should be a top priority, in addition to boosting exercise self-efficacy.

An early measurement tool developed to assess kinesiophobia is the Tampa Scale of Kinesiophobia (TSK) (15, 16). In 2012, Bäck et al. developed a specific scale suitable for assessing the level of kinesiophobia in patients with CHD based on the original TSK scale, known as the Tampa Scale for Kinesiophobia Heart (TSK-SV Heart) (17). In recent years, Chinese scholar Lei and his colleagues translated it into Chinese and validated it in CHD. The Chinese version of the TSK-SV Heart scale has strong reliability and validity, with a split-half reliability of 0.792 and a Cronbach's alpha coefficient of 0.859, and has been popularised in the study of kinesiophobia (18). A cross-sectional study conducted by Ding utilised the TSK-SV Heart to investigate kinesiophobia in 400 patients with heart disease and achieved more significant results (19). Knapik et al. created the Kinesiophobia Causes Scale (KCS) to examine the root causes of passive movement in adults, highlighting that kinesiophobia arises because of a mix of physiological and psychological variables. In 2020, Zhu et al. (20) “Chinese-ised” it and tested its reliability in patients with chronic low back pain, proving that the scale can be used as a valid tool for evaluating the causes of kinesiophobia in China. However, the KCS scale has not yet been validated in patients with heart disease. Therefore, the Chinese version of the TSK-SV Heart scale is selected in the current study to measure the severity of kinesiophobia in post-PCI patients.

Kinesiophobia can be effectively treated in patients with low back pain by a technique called “graded exposure,” which refers to a gradual acceptance of exercise to gradually remove fear (21). The idea of graded exercise has been partially used in exercise-based CR programmes in patients with cardiac infarction (22). According to most guidelines, a regular exercise rehabilitation workout is necessary for patients after PCI. A psychological and behavioural intervention technique, known as cognitive behavioural intervention, is inspired by cognitive behavioural theory. Interventions guided by this theory include assessment of kinesiophobia, education on kinesiophobia, progressive training, and staged rehabilitation. However, these interventions may not be appropriate for patients in phase II of CR (which is typically carried out within 1–6 months of hospital discharge, when patients are at home). With the application of telemedicine, research has found that home-based CR has the same cardiovascular benefits as CR in hospital (23), which can be used as a prominent supplement or alternative. Nurse-led home-based rehabilitation, which improves adherence to rehabilitation, is supposed to help the patients overcome their fears with professional support (24). Furthermore, it has low intervention costs and addresses the fact that most patients do not go to hospital because of time and financial reasons.

Brisk walking is the simplest and most controlled form of CR recommended by national and international guidelines (25, 26). WeChat-based brisk walking refers to the use of a social networking platform by medical experts to direct participants in their continuous rehabilitation to boost their exercise self-efficacy, reduce kinesiophobia, and improve illness prognosis. Research (27) has shown that multidisciplinary or exercise-based cardiac telerehabilitation is a safe and cost-effective alternative to centre-based CR in patients with CHD or chronic heart failure.

Network-based brisk walking has the following advantages. First, professional guidance from medical staff provides authority and safety. Moreover, the simplicity of brisk walking is expected to encourage participation and patient compliance. Finally, and importantly, WeChat is the most popular social software in China and its use is widespread. The current study has three aims: first, to investigate whether a WeChat-based intervention could alleviate kinesiophobia; second, to establish whether the path of alleviation is through improving patients’ exercise self-efficacy; and third, to test whether professionally guided brisk walking could help patients’ cardiorespiratory endurance after PCI.

## 2 The study

### 2.1 Participants

**Figure 1:**
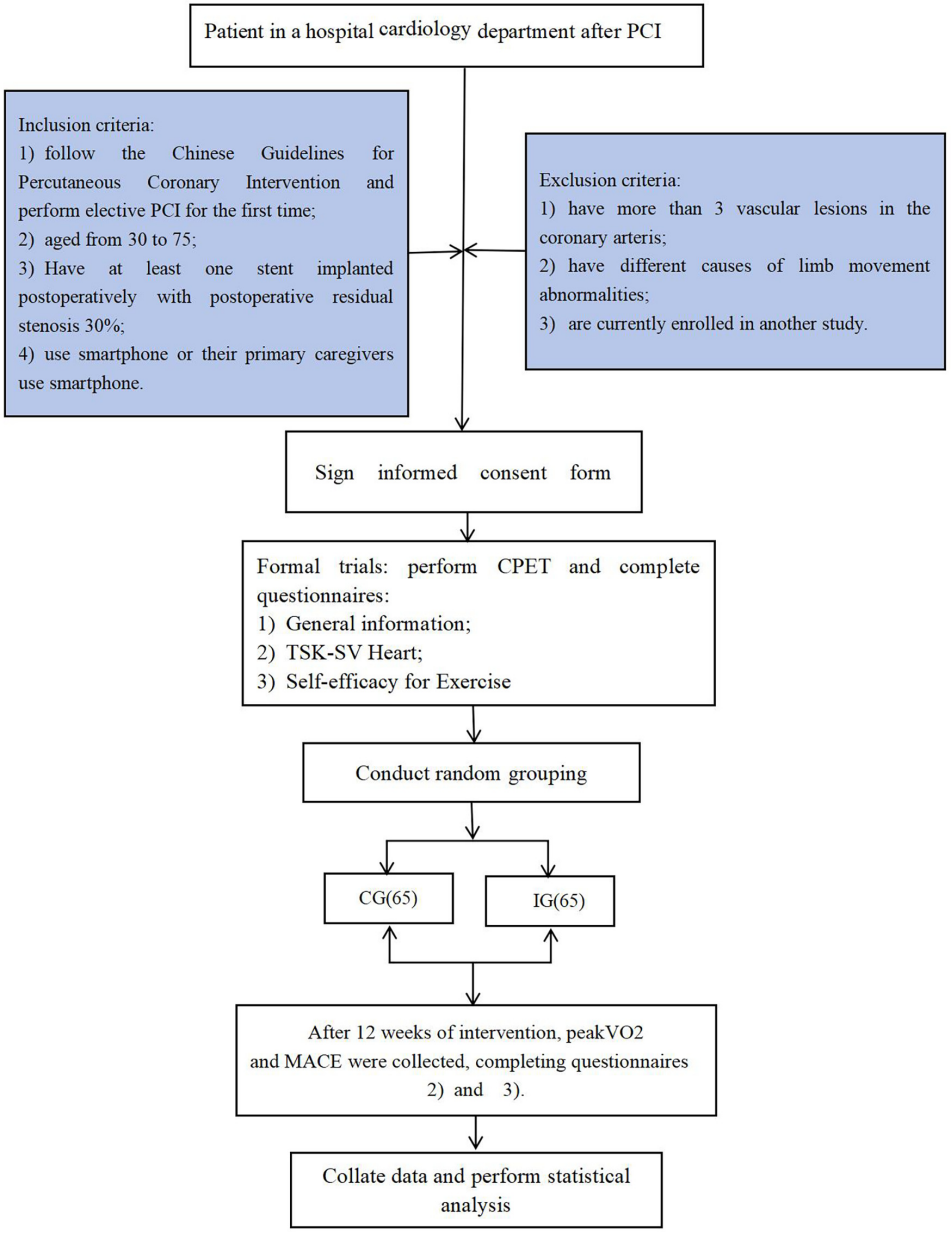
Flow chart.

This study involved a single-blind, prospective, randomised controlled trial. Initially, 137 patients who underwent PCI in our hospital between 1 February 2023 and 31 October 2023 were enrolled (Figure 1). They were divided into an intervention group (IG) and a control group (CG) using the random number table method.

#### 2.1.1 Inclusion criteria

The inclusion criteria were as follows: (1) participants followed the Chinese Guidelines for Percutaneous Coronary Intervention (28) and received elective PCI for the first time (29); (2) patients were aged 30–75 years; (3) patients had at least one stent implanted postoperatively with postoperative residual stenosis of 30% (30); and (4) patients (or their primary caregivers) used a smartphone.

#### 2.1.2 Exclusion criteria

The exclusion criteria were as follows: (1) individuals who had >3 vascular lesions in the coronary arteries; (2) individuals with different causes of limb movement abnormalities; or (3) individuals who were currently enrolled in another study.

#### 2.1.3 Shedding standards

Patients were removed from the study if they (1) had a health condition that made it hard to continue exercising or (2) withdrew for personal reasons.

A total of 137 patients who met the inclusion criteria between 1 February 2023 and 31 October 2023 were included in the study. The sample size is usually calculated based on 5–10 times the number of survey questionnaire items; therefore, the sample size required for this study was 105–170. A total of 130 patients successfully completed a 12-week WeChat-based brisk walking CR programme. There were no statistically significant differences between the two groups of patients with regard to general information (*p* > 0.05).

### 2.2 Method

The home-based rehabilitation team consisted of a chief nurse, a CR physician, a CR therapist, three CR specialist nurses, and two research assistants. All team members had a bachelor's degree or higher, ≥5 years of cardiology healthcare experience, and ≥3 years of CR expertise.

The chief nurse was the team leader and was responsible for team management, coordination, and quality control. The CR physician was responsible for screening patients into groups, formulating exercise prescriptions, and dynamically assessing changes in participants’ conditions. The therapist was responsible for implementing training prescriptions and guiding participants to walk remotely using the Internet. The CR nurse expert was in charge of creating a WeChat group, interacting with participants, scheduling follow-up visits, and constantly documenting the rehabilitation state. The research assistant was responsible for collecting and organising data. All team members underwent rigorous and uniform training before the study.

#### 2.2.1 The intervention group

##### 2.2.1.1 Pre-discharge rehabilitation guidance

Before discharge, the participants’ heart function was assessed using a cardiopulmonary exercise test (CPET) and non-invasive cardiac output. They were then given the TSK-SV Heart scale, Self-Efficacy for Exercise (SEE) scale, and general information questionnaire to complete. After that, the CR physician explained in detail the essentials and precautions for brisk walking, and the CR nurse established a WeChat group and showed them how to utilise the exercise training record sheets. Then, the participants were given the exercise training record sheets and were shown how to record and upload images to the WeChat group with specific information, including resting heart rate, blood pressure, maximum heart rate during exercise, exercise time, and blood pressure monitoring after exercise. Participants’ self-exercise logbooks and telecommunication through WeChat were used to track and maintain their engagement with the exercise programme.

##### 2.2.1.2 Home-based rehabilitation guidance

All IG participants joined the WeChat group, which was hosted by a CR nurse. First, the CR nurse consistently posted the five main CR prescriptions, namely, assistance with exercise, medicine, food, psychological counselling, and quitting smoking. Second, brisk walking was scheduled together with participants. The brisk-walking-at-moderate-intensity training sessions each lasted 30 min and were conducted 3 days a week for the first 4 weeks. Subsequently, exercise intensity was adjusted by increasing the frequency of brisk walking to 5 days a week for the next 8 weeks. Participants followed the CR physician’s walking instructions. For those who had low compliance, the CR nurse would instruct and supervise them via WeChat. Participants uploaded the exercise training record as required. Participants could use a heart rate monitoring application named Physical Examination Treasure (Beijing Aikangkang Jianbao Health Technology Co., Ltd, Beijing, China) on their smartphones to track their heart rate while exercising and upload the information in the WeChat group. The participants measured their blood pressure using calibrated electronic blood pressure monitors (OMRON Corporation, Kyoto City, Japan) provided by the research team (all electronic blood pressure monitors were of the same brand and calibrated before use). Timely feedback and guidance from the team were offered in the WeChat group.

#### 2.2.2 The control group

**Figure 2:**
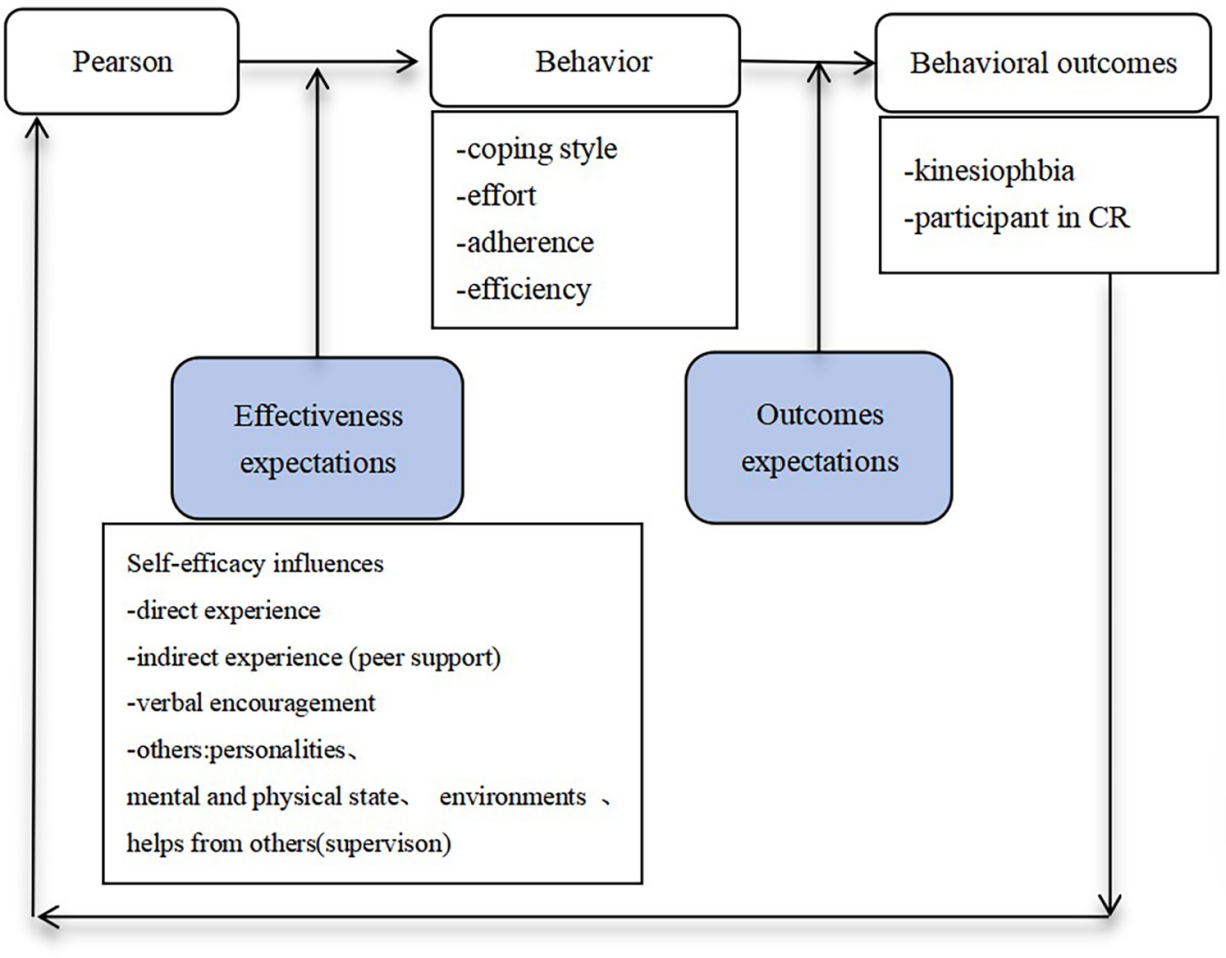
Intervention framework diagram.

Participants in the CG completed the CPET, the general information questionnaire, the TSK-Heart test, and the SEE test on the day of discharge. The CR physician assessed the participant's condition based on the test results. A personalised exercise plan was prescribed, and the importance of adherence was emphasised (see Figure 2, intervention framework diagram).

#### 2.2.3 Evaluation

After 12 weeks of intervention, participants returned to the CR centre and the research assistant performed the data collection. Four patients in the CG and three in the IG dropped out for various reasons, leaving 130 patients (65 in the CG and 65 in the IG). No adverse cardiac events were reported during the intervention period.

### 2.3 Evaluation means

#### 2.3.1 Tampa Scale for Kinesiophobia Heart

The TSK-SV Heart is a specialised scale designed to assess kinesiophobia in patients with CHD. It has four dimensions and 17 items: perception of danger (four items), avoidance of movement (four items), fear of movement (five items), and dysfunction (four items). The items were rated on a 4-point Likert scale in the range of 1–4, with items 4, 8, 12, and 16 having reversed scores, going from “strongly disagree” to “strongly agree.” A high degree of kinesiophobia is indicated by a score of ≥37. This study used a translated Chinese version of the scale, with an overall Cronbach's alpha coefficient of 0.859, a split-half reliability of 0.792, and test–retest reliability of 0.792, demonstrating good reliability and validity (31).

#### 2.3.2 Self-Efficacy for Exercise scale

The SEE scale, developed by Resnick and Jenkins, was used to quantify and reflect participants’ self-evaluation of exercise capacity. The study used the Chinese version, which had nine items and was translated and “Chinese-ised” by Lee et al. (32). A 10-point Likert scale was applied, with a maximum score of 90 points and a range of “no confidence” to “very confident.” The higher the score, the higher the patient's confidence in overcoming obstacles and maintaining a regular exercise regimen. A strong sense of self-efficacy was defined as having a SEE score of ≥50. The scale has a content validity of 0.90 and a Cronbach's alpha of 0.75.

#### 2.3.3 Cardiopulmonary exercise testing and peak oxygen uptake

As an objective evaluation of cardiac function, CPET is frequently used to guide exercise rehabilitation, predict prognosis, and assess rehabilitation outcomes. It can also serve as a foundation for creating exercise prescriptions (33). Peak VO_2_ (the peak kilogram oxygen uptake) is the gold standard for assessing aerobic exercise capacity and predicting prognosis because it shows the cardiac output and reserve function of the heart (34). Peak VO_2_ was the primary CPET indicator used in this study: higher scores denote stronger cardiovascular endurance and reserve function in the patients. Before the test, a MasterScreen CPX device (Jaegar, Bavaria, Germany) was used to measure the participants’ height and weight while they were wearing sportswear and barefoot. Then, participants were seated on a power bicycle with a no-load warm-up for 3 min, and the exercise load was incremented during the test. The CR physician instructed the participants to maintain a bicycle speed of 60–70 rpm until they were exhausted. The test was stopped right away if participants experienced dyspnoea, chest pain, or any other uncomfortable symptoms.

### 2.4 Patient and public involvement

Patients are the main consideration in trial design, subject recruiting, and data exchange and were prioritised as far as possible. A patient involvement group was established at the outset of the study. During the meeting, the patient–public involvement group was informed about how the study would be conducted. Ample time was spent communicating with patients about constructing the intervention programme. After that, a pre-trial was conducted by randomly selecting several patients to gather their opinions and feelings. The research findings will be shared with participants and the public through various channels, including hospital social media accounts and academic lectures.

### 2.5 Statistical analysis

The SPSS 26.0 statistical software package was used for statistical analyses of the data. All data were expressed as mean ± SD or median or percentages (for categorical variables). The Shapiro–Wilk test was used to assess normality. At baseline, the groups were compared using the independent two-sample *t*-test or chi-square test. To compare groups in the follow-up data, the dependent two-sample *t*-test was employed. The matched samples *t*-test was used for within-group comparisons, provided normality was met. If not, the Mann–Whitney *U*-test was used. An intention-to-treat analysis was performed on the primary outcome (kinesiophobia). Pearson correlation coefficients (*P*) were calculated between kinesiophobia and exercise self-efficacy at 12 weeks. A probability level of *p* ≤ 0.05 was considered significant.

## 3 Results

### 3.1 General information

Table 1 shows that there were no significant changes (*p* > 0.05) in any of the following variables between the two groups: gender, age, marital status, occupation, body mass index (BMI), health insurance type, history of coronary hypertension, or co-morbidity. Most of the patients in the population were aged >60 years (mean age 61.42 ± 0.78 years), with unstable angina as their primary ailment (116/130, 89.23%). They had higher body mass indices (mean BMI 23.57 ± 2.80 kg/m^2^) and worked in the brain sciences (65/130, 50.0%). Baseline characteristics were comparable between the groups.

**Table 1:** Comparison of general information of participants in the two groups (*n* = 130).

Variables		The control group (*n* = 65)	The intervention group (*n* = 65)	*t/χ* ^2^	*p*
Gender (%)	Male	53 (81.54)	59 (90.77)	2.321	0.128
	Female	12 (18.46)	6 (9.23)		
Age (years, $\bar{x}\pm s$)		61.35 ± 9.18	62.32 ± 9.63	−0.589	0.558
Marital status (%)	Married	47 (72.31)	42 (64.62)	0.891	0.345
	Unmarried	18 (27.69)	23 (35.39)		
Occupation (%)	Physical work	18 (27.69)	20 (30.77)	2.305	0.316
	Brain work	30 (46.15)	35 (58.85)		
	Other	17 (26.15)	10 (15.38)		
Medical insurance (%)	Provincial health insurance	6 (9.23)	9 (13.85)	1.412	0.494
	City health insurance	50 (76.92)	44 (67.69)		
	Resident health insurance	9 (13.85)	12 (18.46)		
BMI (kg/m^2^, $\bar{x}\pm s$)		24.05 ± 3.09	23.69 ± 2.52	0.711	0.478
Type of CAD (%)	Stable angina	5 (7.69)	9 (13.85)	1.281	0.258
	unstable angina	60 (92.3)	56 (86.15)		
History of hypertension (%)	Yes	18 (27.69)	27 (41.54)	2.753	0.097
	No	47 (72.31)	38 (58.46)		
Number of combined diseases (%)	0–3	32 (49.23)	24 (36.92)	4.469	0.107
	3–6	18 (27.69)	30 (46.15)		
	>6	15(23.08)	12 (18.46)		

BMI, body mass index; CAD, coronary artery disease.

### 3.2 The Tampa Scale for Kinesiophobia Heart score and Self-Efficacy for Exercise score before and after the intervention

The results showed that the two groups of TSK-SV Heart scores did not differ significantly before the intervention (*t* = −0.835, *p* > 0.05). However, after 12 weeks of intervention, the TSK-SV cardiac function scores in both groups decreased, and the intervention group scored lower than the CG, with a large difference (*t* = 7.033, *p* < 0.001) (Table 2).

**Table 2:** Comparison of TSK-SV Heart between the two groups (mean ± SD, *n* = 130).

Groups	At discharge	12 weeks after discharge	*t*	*p*
The control group	46.27 ± 5.87	45.50 ± 4.26	0.856	0.394
The intervention group	47.05 ± 4.71	40.95 ± 3.08	8.738	<0.001
*t*	−0.835	7.033	—	—
*p*	0.405	<0.001	—	—

The results showed no significant change in SEE scores in both groups before the intervention (*t* = −1.337, *p* > 0.05). There was no significant change in SEE score before and after intervention in the control group. After 12 weeks of intervention, the mean SEE score (43.77 ± 4.62) was significantly higher than the mean score before the intervention (36.95 ± 5.13; *t* = −7.964, *p* < 0.001). The SEE score of the intervention group was higher than that of the control group after 12 weeks of intervention. (*t* = −9.186, *p* < 0.001) (Table 3).

**Table 3:** Comparison of SEE between the two groups (mean ± SD, *n* = 130).

Groups	At discharge	12 weeks after discharge	*t*	*p*
The control group	35.50 ± 7.08	35.81 ± 5.24	−0.283	0.777
The intervention group	36.95 ± 5.13	43.77 ± 4.62	−7.964	<0.001
*t*	−1.337	−9.186	—	—
*p*	0.183	<0.001	—	—

There was no significant correlation of change in SEE with change in TSK-SV Heart (Pearson *P* = −0.007, *p* = 0.934). However, a significant correlation of SEE with TSK-SV Heart (*P* = −0.449, *p* < 0.001) at 12 weeks was found.

The results showed no difference between the two groups before the intervention (*t* = 1.598, *p* = 0.112). In the intervention group, after 12 weeks of intervention, the mean peak VO_2_ (24.24 ± 4.43) was significantly higher than that before the intervention (21.10 ± 3.73; *t* = −4.371, *p* < 0.001). The peak VO2 of the IG was higher than that of the CG after 12 weeks of intervention (*t* = −6.184, *p* < 0.001) (Table 4).

**Table 4:** Comparison of peak VO_2_ in the two groups (mean ± SD, *n* = 130).

Groups	At discharge	12 weeks after discharge	*t*	*p*
The control group	20.09 ± 3.47	20.17 ± 2.92	−0.142	0.887
The intervention group	21.10 ± 3.73	24.24 ± 4.40	−4.371	<0.001
*t*	1.598	−6.184	—	—
*p*	0.112	<0.001	—	—

### 3.3 Report on the test safety

The fact that no adverse cardiovascular events happened throughout the entire intervention indicates that the trial was safe. To protect the participants’ safety, a very thorough adverse event protocol was created.

## 4 Discussion

Kinesiophobia may contribute to less physical involvement in a considerable portion of patients with CHD. After 12 weeks of intervention, the kinesiophobia scale scores of the IG reduced considerably, demonstrating the benefit of the WeChat intervention (35). Through WeChat, participants received positive comments and encouragement from health workers as well as moral support from their peers. Health workers are individuals they trust, and peers are those with whom they jointly fight the ailment. According to Bandura's theory, positive feedback and encouragement are necessary to increase self-efficacy (36); the theoretical framework is shown in Figure 2. We supposed that the WeChat-based intervention significantly reduced kinesiophobia by enhancing self-efficacy. Self-efficacy is defined as the patients’ conviction in their own ability to carry out or sustain a habit, which is an important indication of exercise adherence (37). The primary theoretical component elements of self-efficacy are direct experience (e.g., performance achievement), indirect experience, verbal encouragement, and others (e.g., physiological and physical conditions). The verbal encouragement intervention to improve self-efficacy is characterised by growing one's confidence via positive feedback and persuasion, whereas indirect experience is characterised by support from peers. The IG participants exhibited a substantial increase in exercise self-efficacy after the intervention, which is similar to the study by Wang et al. (38). Domestic studies have shown that patients with higher levels of self-efficacy have better exercise compliance (39, 40). Patients with stronger self-efficacy are more enthusiastic about exercising and are less likely to quit, which is the final purpose of any intervention to encourage patients to take CR after PCI.

Brisk walking plays a positive role in improving athletic ability after PCI (41, 42). It is also the most accepted form of exercise for elderly people in China because of its convenience, low cost, and home-based nature. A study has shown that brisk walking for 30 min per day for approximately 3 months improved exercise endurance in patients with CHD (43). Previous studies have focused on the overall interventions and outcomes (44, 45); however, there remains a concern that brisk walking cannot improve patient compliance. The results of the present study showed that after 12 weeks of intervention, peak VO_2_ in the IG significantly increased compared with the CG and pre-IG, which is consistent with most studies in China and abroad (46, 47). In our study, with the use of WeChat, the home-based CR intervention successfully raised cardiopulmonary endurance in patients after PCI. These outcomes demonstrated that WeChat-based brisk walking improved participants’ adherence because it did not have commuting difficulties and scheduling issues compared with conventional brisk walking, thus raising their cardiopulmonary endurance. In addition, older patients who lack social support and are in worse physical condition are more likely to quit the rehabilitation (27); however, WeChat-based brisk walking plays a crucial role in monitoring their exercise. Through professional guidance, it helps patients develop a daily exercise habit in a way that is both effective and comfortable for them, as expected. Moreover, the WeChat-based brisk walking for post-PCI patients is holistic and continuous. Nurses oversee the entire process, monitoring participants’ conditions and providing guidance on maintaining an optimal heart rate. They also issue alerts when a participant’s heart rate approaches its maximum during exercise. Ensuring safety is crucial to the successful implementation of WeChat-based brisk walking post-PCI patients. In this study, no patient trials involved significant safety risks.

The WeChat-based brisk walking rehabilitation programme provides greater convenience for patients facing financial difficulties or living in remote areas. It may offer more significant benefits and a better cost–benefit ratio than conventional methods (48). In addition, this novel intervention may help patients sustain long-term healthy behaviours.

However, our study has some. First, it included only patients from the cardiology department of a single hospital, resulting in limited sample representativeness. Future research should expand the scope by conducting a multicentre randomised controlled trial. Second, because of limited funding and personnel resources, the intervention only lasted 12 weeks, preventing an assessment of its long-term effects.

## 5 Conclusion

The WeChat-based brisk walking CR management programme enhances exercise self-efficacy and reduces kinesiophobia through three modalities: medical staff support, positive feedback, and encouragement. It also improves patients’ adherence to exercise, leading to better cardiorespiratory endurance. The WeChat-based brisk walking intervention is both feasible and acceptable, overcoming time and space limitations to support disease management in post-PCI patients. By leveraging established online social media platforms, nurses can share health education materials and engage with patients remotely, which will accelerate the development of e-health.

## Data Availability

The original contributions presented in the study are included in the article/Supplementary Material, further inquiries can be directed to the corresponding author.
